# Amelioration of Disease Manifestations by Saffron Extract in a Mouse Model of Collagenase-Induced Osteoarthritis

**DOI:** 10.3390/ijms27104165

**Published:** 2026-05-07

**Authors:** Blagovesta Todorova, Nikoleta Dyakova, Petya Ganova, Andrey Tchorbanov, Nikolina Mihaylova

**Affiliations:** 1 Department of Immunology, Institute of Microbiology, Bulgarian Academy of Sciences, 1113 Sofia, Bulgaria; boneva_b@microbio.bas.bg (B.T.); nikoleta.dyakova@microbio.bas.bg (N.D.); pganova@gmail.com (P.G.); tchorban@microbio.bas.bg (A.T.); 2 Center of Competence “Fundamental, Translational and Clinical Investigations on Infections and Immunity”, 1000 Sofia, Bulgaria

**Keywords:** collagenase, experimental osteoarthritis, inflammation, saffron

## Abstract

Osteoarthritis (OA) is a degenerative, age-related joint disease involving bone remodeling and damage to articular cartilage. OA is the most common form of arthritis, causing pain, swelling, stiffness, and reduced mobility. Given the limited efficacy of current therapies, there is growing interest in natural compounds with anti-inflammatory and immunomodulatory properties. *Crocus sativus* L., known as saffron, contains more than 150 biologically active compounds with proven antioxidant and anti-inflammatory effects, making it a promising candidate for modulating OA-related processes. The aim of the study was to evaluate the effects of saffron extract on immune cell function, osteoclast differentiation, and joint pathology in collagenase-induced osteoarthritis (CIOA) mouse model. OA was induced by intra-articular injection of Collagenase type IA, followed by daily treatment with saffron extract for 30 days. Flow cytometry, apoptosis, Western blot and proliferation assays were performed to analyze the phenotype and activity of bone marrow and synovium cells, as well as histological evaluation of joint tissues. Saffron therapy promoted an anti-inflammatory immune profile, reduced T-cell apoptosis and proliferation, and inhibited osteoclast differentiation. These changes were accompanied by improved histological manifestations of the joints. Overall, the findings suggest that saffron may modulate key inflammatory and cellular mechanisms involved in OA, although further research is needed to confirm its therapeutic relevance in humans.

## 1. Introduction

Osteoarthritis (OA) is a chronic, debilitating, age-related joint disease affecting nearly 606.5 million people worldwide, or 7.6% of the global population [1,2,3,4,5,6]. This increased burden appears to be more noticeable among younger individuals-52.3% of the reported cases of OA occurred in individuals under the age of 55 [7]. The most significant risk factors that contribute to the higher incidence of OA include obesity and injuries, as well as structural abnormalities, behavioral factors, physical activity, and metabolic disorders [8,9,10]. It is predicted that by 2050, the number of people living with various forms of OA will reach 1 billion people, which would be a serious challenge for healthcare systems [11]. Furthermore, according to the systematic literature review summarizing 20 years’ data, OARSI (The Osteoarthritis Research Society International) summarizes an increased risk of mortality in OA [12].

OA is a slow progressive condition, and its physical manifestations include pain, stiffness, locomotor restriction, bone enlargement, swelling of the affected joints, impaired function, and wear and tear of the joints. All this leads to bone remodeling, osteophyte formation, fibrosis and hyperplasia of the synovial membrane, and damage or loss of articular cartilage [13,14]. Pathological changes in affected joints are highly dependent on genetic, metabolic, biochemical and biomechanical factors, followed by key activation of the inflammatory response, subchondral bone degradation, osteoclastogenesis, chondrocytes, synoviocytes and bone marrow [15,16,17,18]. Understanding these multifaceted interactions is key to the search for therapeutic approaches.

AO is recognized as a low-grade inflammatory disease. Among the cell types involved, macrophages are highly adaptive immune cells with a central role in bone loss in inflammatory bone diseases and OA, and dominate the response [19,20]. Depending on the signals, they can shift between pro-inflammatory (M1) and anti-inflammatory (M2) phenotypes [21]. Interactions with articular chondrocytes and intense infiltration of immune cells into the synovium contribute to a highly reactive inflammatory environment [22,23,24]. These processes, along with osteoclast differentiation, are driven by factors such as cytokines, adipokines, abnormal metabolites, acute phase reactants, vitamin D deficiency, and impaired microRNA metabolism [25,26,27].

There is no effective cure for OA, partly due to the lack of a proven algorithm for the initiation and progression of the pathological mechanism of the disease. A variety of classic drugs are used for the therapy of OA, such as acetaminophen, nonsteroidal anti-inflammatory drugs (NSAIDs), opioid analgesics, serotonin-norepinephrine reuptake inhibitors (SNRIs), intra-articular injection, chondrogenesis inducers, osteogenesis inhibitors, matrix degradation inhibitors, apoptosis inhibitors, and anti-inflammatory cytokines [13,28]. Although these therapies are commonly used to treat pain, their long-term use requires careful monitoring due to the side effects.

Another therapeutic approach used is autologous bone marrow-derived mesenchymal stem cells (BM-MSCs) and adipose-derived MSCs (AD-MSCs). Although the exact mechanism of their effectiveness is not fully understood, results in clinical practice show benefits in terms of symptoms and functionality [29]. A group of drugs, such as metformin, statins, angiotensin-converting enzyme (ACE) inhibitors, and angiotensin II receptor blockers (ARBs), exhibit a number of pleiotropic effects and reduce inflammation caused by OA, promote cartilage maintenance and repair, and improve the synthesis of cartilage proteoglycans and collagen [30,31,32,33]. The last therapeutic option for treating OA is surgical interventions, but they still depend on access, social, economic, and geographical factors [34]. Therefore, therapeutic design and development of new generations of drugs depend on a better understanding of signaling pathways and key molecules involved in the pathogenesis of OA [35].

Natural products and traditional medicine provide a number of molecules and combinations for long-term use with fewer negative effects. Randomized trials using nutritional supplements such as curcumin and Boswellia serrata suggest their potential anti-inflammatory and analgesic effects [35,36]. One of the most famous plants for its medicinal effects is *Crocus sativus* L., known as saffron. Saffron extract contains more than 150 constituents and biologically active compounds such as crocin and crocetin, two carotenoid pigments responsible for its color; picrocrocin, which provides its aroma and bitter taste; and safranal, a volatile compound responsible for its odor [37]. Numerous studies have demonstrated a number of beneficial biological effects of saffron, including improvement of symptoms of depression and anxiety [38,39,40], its cognitive and neuroprotective benefits [41,42,43], its metabolic effects [44], as well as anti-inflammatory and antioxidant activities [45,46].

Late detection, slow progression of OA in humans and ethical reasons make animal models an alternative option for developmental research, studying pathogenesis and potential therapeutic intervention regarding pathological processes, involved cells and biomolecular markers [26,34]. Inducing disease in the laboratory can be done in four different ways: chemical substances, genetically manipulated mice, surgically weakening the ligaments in the joints, or biochemically [4,47,48]. The collagenase-induced osteoarthritis (CIOA) mouse model is a widely used chemically induced model that mimics key pathological features of human osteoarthritis. Compared to other mouse models (e.g., surgical models), CIOA shows a faster onset and stronger inflammatory component. In a previous study, we demonstrated the effectiveness of the CIOA model. All experimental animals developed well-described pathological symptoms characterizing osteoarthritis, providing data for defining and monitoring the stages of the disease and the interaction between cells and tissues [49].

The aim of the present study was to investigate the potential of bioactive natural products, such as saffron extract, to ameliorate OA symptoms by reducing pro-inflammatory immune cell subtypes and increasing antioxidant capacity. Research into these aspects may reveal new mechanisms of action and improve its therapeutic potential.

## 2. Results

### 2.1. Treatment of Mice with Saffron Extract

**Figure 1:**
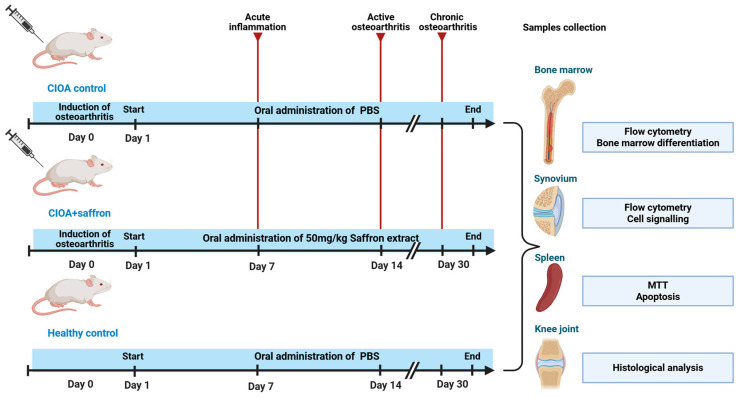
Scheme of the treatment protocol and analyses.

Male BALB/c mice (10–12 weeks old) were challenged with collagenase Type IA to induce CIOA by intra-articular injection. On the day after the collagenase challenge, the animals were randomly assigned into two groups—the first group (CIOA + saffron) was administered *Crocus sativus* extract, and the second group (CIOA) was treated with PBS only. Another control group of intact healthy mice (Healthy) was fed with PBS. All animals were fed daily per os for one month, and the effect of saffron extract was studied. A summarized scheme representing therapy with saffron extract is shown in Figure 1.

### 2.2. Phenotyping of Bone Marrow and Synovial Cells

**Figure 2:**
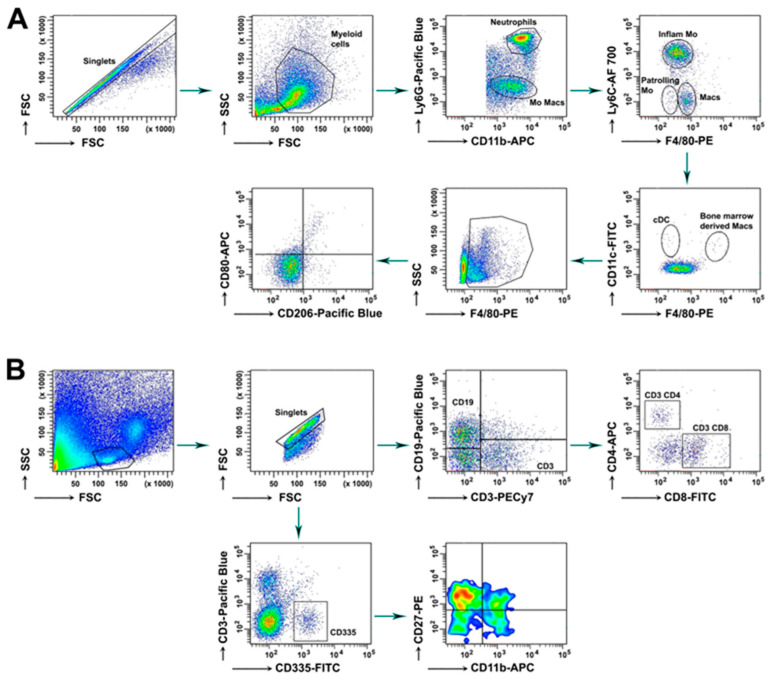
Gating strategy for myeloid (**A**) and lymphoid (**B**) cell populations in bone marrow and synovium.

As already shown, the progression of osteoarthritis develops silently and can be divided into three main periods: acute (seven days after induction), active (14 days after induction) and chronic (30 days after induction). To provide a clearer picture of cell-to-cell communication during disease development and the effects of the tested extract, we analyzed by FACS the diversity of immune cell populations in the bone marrow (BM) and in the synovium in these three stages of CIOA development. Myeloid lineage subpopulations and major lymphocyte populations (Figure 2) were analyzed in the BM as follows: CD11b^+^ F4/80^−^ Ly6C^hi^ and CD11b^+^ F4/80^−^ Ly6C^lo^ monocytes; the bone-marrow derived macrophages (CD11c^+^ F4/80^hi^ Ly6C^low^), the neutrophil population (CD11b^+^ Ly6G^+^ F4/80^−^), M1 (F4/80^hi^ CD80^+^), M2 (F4/80^hi^ CD206^+^) and M1/M2 (F4/80^hi^ CD80^+^ CD206^+^) macrophages. 

**Figure 3:**
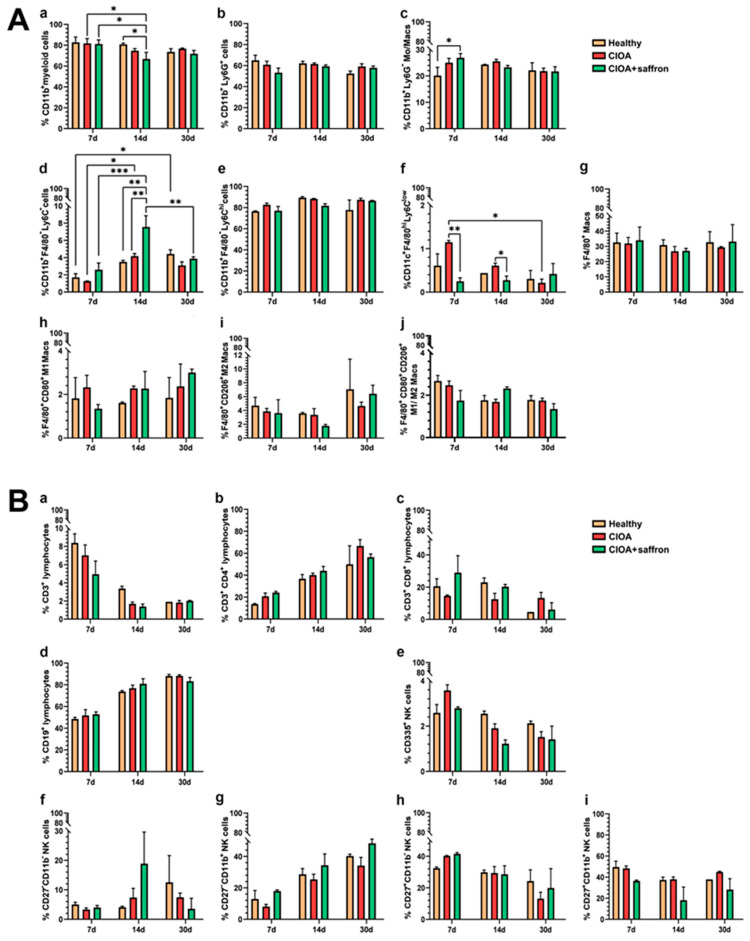
Distribution of myeloid (**A**) and lymphoid (**B**) cell populations in the bone marrow of CIOA animals isolated on days 7, 14 and 30 after disease onset. Mice were treated with 50 mg/kg saffron extract. (**A**) The extracted results of CD11b^+^ myeloid cells (**a**), CD11b^+^Ly6G^+^F4/80^−^ neutrophil cell population (**b**), CD11b^+^Ly6G^−^ Mo/Mac (**c**), CD11b^+^ F4/80^−^ Ly6C^hi^ and CD11b^+^F4/80^−^Ly6C^low^ monocytes (**d**,**e**), CD11c^+^F4/80^hi^Ly6C^low^ bone-marrow derived macrophages (**f**), F4/80^+^ macrophages (**g**), M1 (F4/80^hi^ CD80^+^) (**h**), M2 (F4/80^hi^ CD206^+^) (**i**), and M1/M2 (F4/80^hi^ CD80^+^ CD206^+^) (**j**) macrophages from all experiments are presented graphically; (**B**) The extracted results of CD19+ lymphocytes (**a**), CD3^+^ lymphocytes (**b**), CD3^+^CD4^+^ T cells (**c**), CD3^+^CD8^+^ T cells (**d**), CD3^−^CD335^+^NK cells (**e**), CD27^−^CD11^b-^NK cells (**f**), CD27^−^CD11b^+^ NK cells (**g**), CD27^+^CD11b^−^ NK cells (**h**), and CD27^+^CD11b^+^ NK cells (**i**) from all experiments are presented graphically. Data are presented as mean ± SD (*n* = 5) and were analyzed by the two-way ANOVA test followed by Tukey’s multiple comparison test (* *p* < 0.05; ** *p* < 0.01; *** *p* < 0.001).

Analyzing myelopoiesis in the different stages of the disease, no significant sustained effect was observed in the experimental groups. A slight decrease without widespread suppression of myelopoiesis in the CIOA + saffron group was found on day 14 (Figure 3A(a)). We did not observe significant changes in the neutrophil population (Figure 3A(b)) and the CD11b^+^ F4/80^−^ Ly6C^hi^ monocytes (Figure 3A(e)). The proportion of CD11b^+^ Ly6G^−^ monocytes was slightly increased on day 7 after collagenase challenge, after which the same cells normalized in the CIOA + saffron group (Figure 3A(c)). The most noticeable change is observed in the population of so-called patrolling monocytes (CD11b^+^ F4/80^−^ Ly6C^lo^). The CIOA + saffron group showed significantly increased levels of this cell population compared to the control groups after 14 days of therapy (Figure 3A(d)). These non-classical cells explore blood vessels and initiate tissue repair and resolution of inflammation, often acting as precursors to tissue-resident cells. In the chronic phase of CIOA (day 30), the same monocyte population was reduced to levels comparable to healthy controls. The increase in CD11b^+^ F4/80^−^ Ly6C^−^ cells observed in healthy animals at later time points likely reflects physiological myeloid maturation and the expansion of non-inflammatory dendritic-like populations (Figure 3A(d)).

As expected, seven days after CIOA induction, a statistically significant increase in the bone-marrow-derived macrophages (CD11c^+^ F4/80^hi^ Ly6C^low^) was observed in the CIOA group. At the same time, the number of these macrophages remained very low in the bone marrow of the CIOA + saffron group throughout the entire studied period (Figure 3A(f)). No significant differences were found in the BM populations of M1, M2 and M1/M2 macrophages between the experimental groups (Figure 3A(h,i,j)). 

Analysis of lymphocyte populations in the BM of the experimental groups showed no statistically significant differences regarding T cell subpopulations (Figure 3B(a–c)) and B cells (Figure 3B(d)) nevertheless the therapy protocol. Intra-articular injection with collagenase altered the CD3^−^CD335^+^ NK cell population in the BM. Administration of the studied extract did not significantly affect the percentage of the different NK cell subpopulations, although several minor changes were observed, especially in the population of immature and inactive phenotype (CD27^−^CD11b^−^ NK cells) (Figure 3B(e–i)).

**Figure 4:**
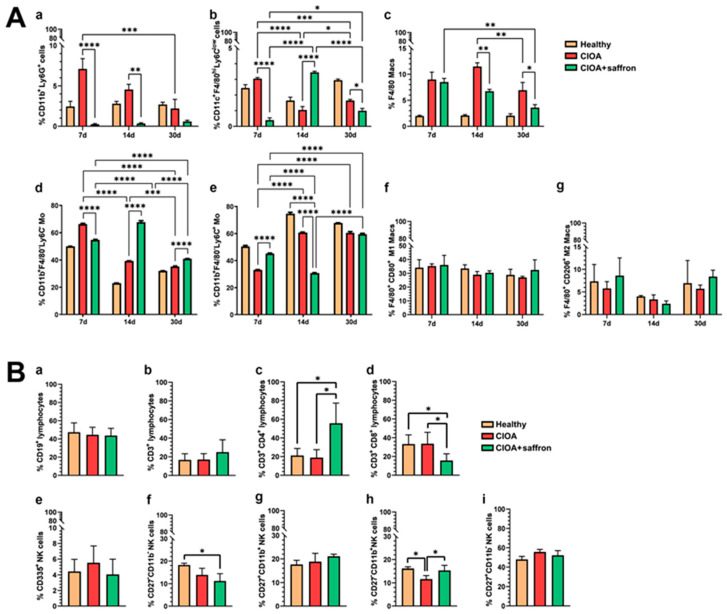
Distribution of myeloid (**A**) and lymphoid (**B**) cell populations in synovium of CIOA animals isolated on days 7, 14 and 30 after disease onset. Mice were treated with 50 mg/kg saffron extract. (**A**) The extracted results of CD11b^+^ Ly6G^+^ F4/80^−^ neutrophil cell population (**a**), CD11c^+^ F4/80^hi^ Ly6C^low^ bone-marrow derived macrophages (**b**), F4/80^+^ macrophages (**c**), CD11b^+^ F4/80^−^ Ly6C^−^ and CD11b^+^ F4/80^−^ Ly6C^+^ monocytes (**d**,**e**), M1 (F4/80^hi^ CD80^+^) (**f**), and M2 (F4/80^hi^ CD206^+^) (**g**) macrophages from all experiments are presented graphically; (**B**) The extracted results of CD19^+^ lymphocytes (**a**), CD3^+^ lymphocytes (**b**), CD3^+^CD4^+^ T cells (**c**), CD3^+^CD8^+^ T cells (**d**), CD3^−^CD335^+^ NK cells (**e**), CD27^−^CD11b^−^ NK cells (**f**), CD27^+^CD11b^+^ NK cells (**g**), CD27^−^CD11b^+^ NK cells (**h**), and CD27^+^CD11b^−^ NK cells (**i**) from all experiments are presented graphically. Data is represented as mean ± SD (*n* = 5) and were analyzed by the two-way ANOVA test followed by Tukey’s multiple comparison test (* *p*< 0.05; ** *p*< 0.01; *** *p* < 0.001; **** *p* < 0.0001).

To monitor the effects of saffron extract on the systemic-local axis, inflammatory responses were followed up in the synovium of all experimental groups throughout the therapeutic period. Induction of OA with collagenase results in a strong response from the neutrophil population (CD11b^+^ Ly6G^+^) in the synovium. Seven days after OA induction, the CIOA group showed a high proportion of neutrophils, which decreased over the study period. In contrast, animals treated with 50 mg/kg saffron extract showed a significant reduction in the CD11b^+^ Ly6G^+^ population during the same treatment period (Figure 4A(a)). The population of CD11c^+^ F4/80^hi^ Ly6C^low^ cells in saffron-treated mice was very low on day 7 of starting therapy compared to other control groups. Seven days later, a significant increase in the percentage of the same cells was observed, which decreased again at the end of the observation (Figure 4A(b)). The same cell population in the CIOA control group showed similar behavior to bone marrow. The population of F4/80 macrophages increased in both CIOA groups soon after disease onset, but a significant decrease in the percentage of these cells was observed on day 14 and day 30 in the joints of the CIOA + saffron group compared to untreated CIOA controls (Figure 4A(c)).

Very significant changes were observed in the CD11b^+^ F4/80^−^Ly6C^low^ and CD11b^+^ F4/80^−^Ly6C^hi^ monocyte populations. The CIOA + saffron group showed an increase in the percentage of regulatory-like CD11b^+^ F4/80^−^ Ly6C^low^ monocytes and a decrease in the inflammatory CD11b^+^ F4/80^−^ Ly6C^hi^ population in the active phase (day 14) of OA development. Opposing results were found in the CIOA group in the same phase of the disease (Figure 4A(d,e)). No statistically significant differences were measured between animal groups according to M1 and M2 macrophage distribution (Figure 4A(f,g)).

The analysis of the lymphocyte populations presented in the synovium of experimental animals was performed on day 30 after CIOA induction. We could not detect significant differences between animal groups in the populations of CD19 and CD3 lymphocytes in the synovium (Figure 4B(a,b)). A significant increase in CD4^+^ T cells and a decrease in the cytotoxic CD8^+^ T cell population were observed in the CIOA + saffron group compared to control animals (Figure 4B(c,d)). The studied extract did not affect the population of CD335^+^ NK cells (Figure 4B(e)), but analysis of NK cell subpopulations showed an increase in CD27^−^CD11b^+^ mature NK cells and a decrease in CD27^−^CD11b^−^ undifferentiated NK cells compared to the CIOA control group (Figure 4B(f,h)). No significant changes were found in other NK cell subpopulations (Figure 4B(g,i)). 

### 2.3. Analysis of B and T Cell Apoptosis Ex Vivo

**Figure 5:**
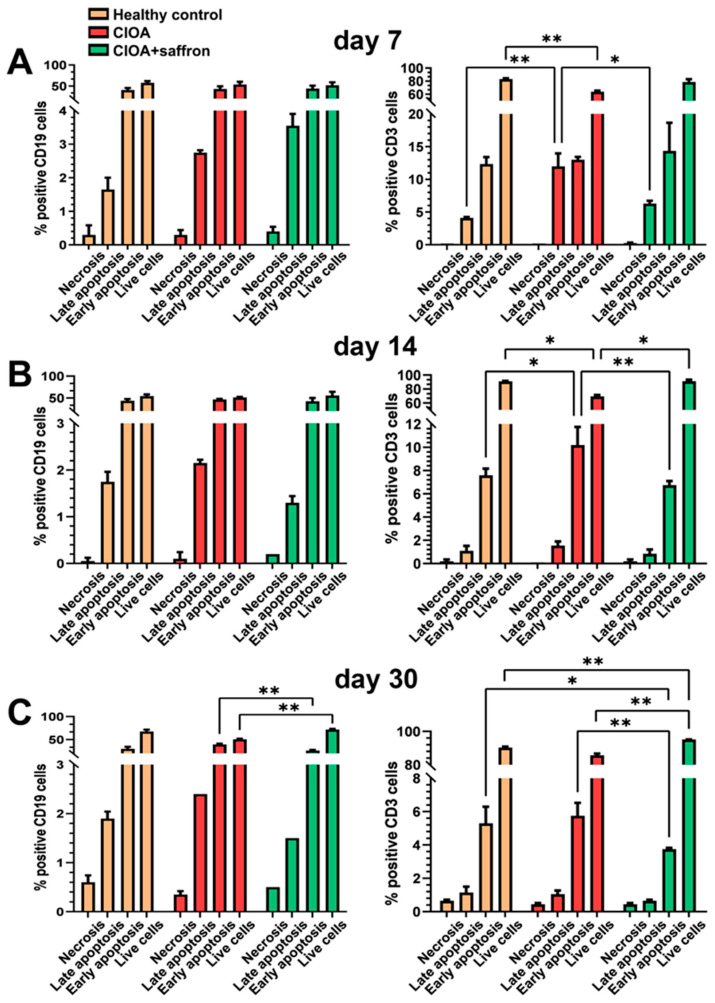
Apoptosis analysis of B- and T-lymphocytes isolated from the spleens of experimental animals with induced OA treated with 50 mg/kg saffron extract. (**A**) Analyzed on day 7 after collagenase injection; (**B**) Analyzed on day 14 after collagenase injection; (**C**) Analyzed on day 30 after collagenase injection. Results are presented as mean ± SD (*n* = 5). The data were analyzed using two-way ANOVA followed by Tukey’s multiple comparison test (* *p* < 0.05; ** *p* < 0.01).

B and T lymphocytes, as part of the adaptive immune system, play an important role in the development of OA. To expand the information on their importance in the pathology of the CIOA model, we have used splenocytes from all experimental animals and followed the impact of the saffron extract on T and B cell apoptosis. Seven days after osteoarthritis induction, an insignificant increase in late B cell apoptosis was observed in both groups of animals with CIOA, treated or untreated with saffron, compared to healthy controls (Figure 5A, left panel). At the same period, elevated levels of late apoptosis and reduced T cell survival were observed in control CIOA animals compared to healthy controls, while saffron treatment maintained levels comparable to healthy mice (Figure 5A, right panel).

On day 14 (active phase) after CIOA induction, significantly reduced levels of early apoptosis and improvement in overall T cell survival were found in the CIOA + saffron group compared to untreated CIOA mice (Figure 5B, right panel), and no significant changes were measured in B cells’ apoptosis (Figure 5B, left panel).

In the chronic phase (day 30) of osteoarthritis progression, the data obtained confirmed the protective effect of saffron extract by reproducing the result of reduced early apoptosis and improved overall T cell survival (Figure 5C, right panel). Here, a statistically significant reduction in early apoptosis and increased B cell survival was found in saffron-treated animals compared to untreated CIOA mice (Figure 5C, left panel).

### 2.4. Ex Vivo Analysis of the Effects of Saffron Extract Treatment on Cell Proliferation

**Figure 6:**
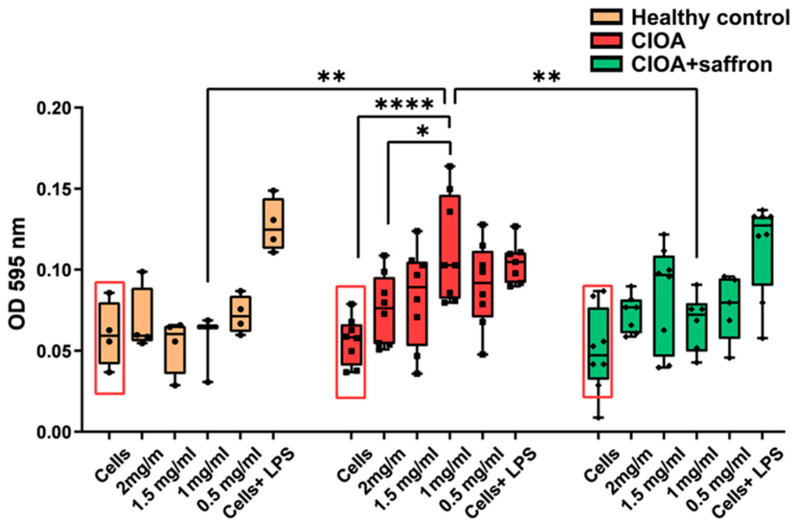
Ex vivo study of splenocyte proliferation cultured for 72 h in the presence of different concentrations of saffron extract. Data are presented as mean ± SD (*n* = 5). The data were analyzed using two-way ANOVA followed by Tukey’s multiple comparison test (* *p* < 0.05; ** *p* < 0.01; **** *p* < 0.0001).

Experiments to evaluate the effects of saffron extract on cell proliferation and survival were performed after 72 h of incubation of splenocytes isolated from animals in all experimental groups with different concentrations of the extract. Experiments were conducted at the end of the treatment schedule (day 30). No significant differences were observed in the ex vivo proliferative activity of splenocytes in any of the test groups without additional in vitro stimulation (red rectangles, Figure 6). Furthermore, in vitro incubation of splenocytes from healthy Balb/c mice with different concentrations of saffron extract resulted in neither stimulation nor suppression of cell proliferation, compared to stimulation with LPS as a positive control. In contrast, splenocytes isolated from animals with CIOA and treated with PBS only showed a significant dose-dependent stimulatory response when incubated with saffron extract compared to untreated cells from the same group. No significant differences were found between cells incubated ex vivo with saffron extract among the group of CIOA + saffron animals treated in vivo with 50mg/kg extract. We observed a significant reduction in proliferation of the CIOA + saffron group cultured in the presence of 1 mg/mL saffron extract ex vivo, compared to control CIOA mice cultured with the same concentration of the extract (Figure 6). No significant differences were measured after 48 h of incubation with the test extract for all groups.

### 2.5. Analysis of the Effect of Saffron Extract on Osteoclast and Osteoblast Differentiation In Vitro

**Figure 7:**
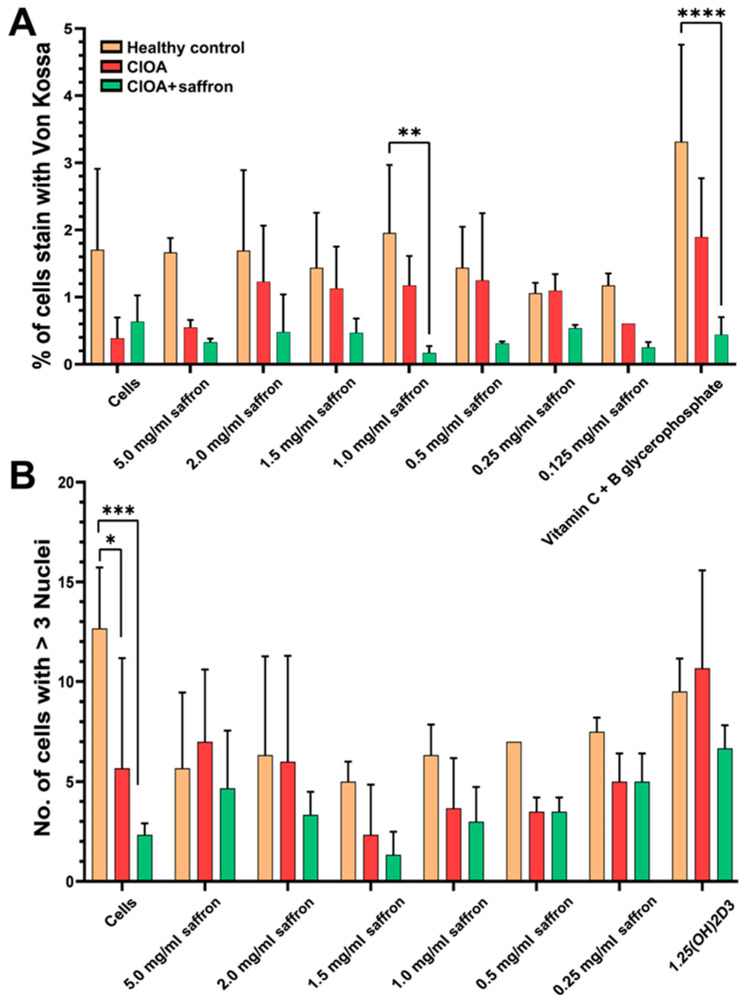
Analysis of the ex vivo differentiation of bone marrow cells into osteoblasts (**A**) and osteoclasts (**B**) in the presence of different concentrations of saffron extract. Graph A represents the percentage of cells stained with Von Kossa and defined as osteoblasts. Graph B represents the number of multinucleated cells and defined as osteoclasts. Data are presented as mean ± SD (*n* = 5) and were analyzed by two-way ANOVA test followed by Tukey’s multiple comparison test (* *p* < 0.05; ** *p* < 0.01; *** *p* < 0.001; **** *p* < 0.0001).

The balance between osteoblastogenesis and osteoclastogenesis is a central component of bone remodeling in OA, and its disruption contributes to disease progression. The ex vivo and in vitro effect of saffron extract on pathological joint remodeling processes was tested using bone marrow cells from experimental animals. Experiments were conducted at the end of the treatment schedule (day 30). Treatment of experimental CIOA mice with saffron extract in vivo did not significantly enhance osteoblast differentiation resulting from ex vivo cultivation of bone marrow cells compared to the control untreated CIOA group (Figure 7A). Surprisingly, in vitro treatment with different concentrations of saffron extract did not affect cell differentiation in the CIOA group treated in vivo with 50 mg/kg saffron. The same trend was observed for the bone marrow cells of the Healthy control group. The additional in vitro stimulation of bone marrow cells with different concentrations of the saffron extract increased the percentage of the cells insignificantly, with higher levels of calcification in the CIOA control group compared to the CIOA + saffron group (Figure 7A).

To evaluate the anti-osteoclastogenic potential of saffron extract, in vitro osteoclast differentiation induced by 1,25-dihydroxyvitamin D3 was performed. The bone marrow cells were incubated in the presence of different concentrations of the tested extract. Control cells were incubated in medium alone to monitor the in vivo effect of the treatment. Saffron extract significantly inhibits the in vivo differentiation of bone marrow osteoclast precursor cells to multinucleated osteoclast cells without in vitro treatment (Figure 7B). Saffron therapy ex vivo did not significantly affect cells from different groups of animals, although the CIOA + saffron group maintained insignificantly lower levels than controls. Although the results were statistically insignificant, it was observed that saffron therapy could restore osteoblast-osteoclast balance by controlling osteoblast activity while simultaneously suppressing osteoclast functionality. 

### 2.6. Effects of Saffron Extract on the Histopathological Changes in the Knee Joint of Animals with CIOA

A detailed histological evaluation of the joint tissues of animals in the three experimental groups was performed. A series of specific histochemical stains was performed to assess the overall histopathological changes, the extent of proteoglycan and glycosaminoglycan loss, and the degree of bone calcification.

**Figure 8:**
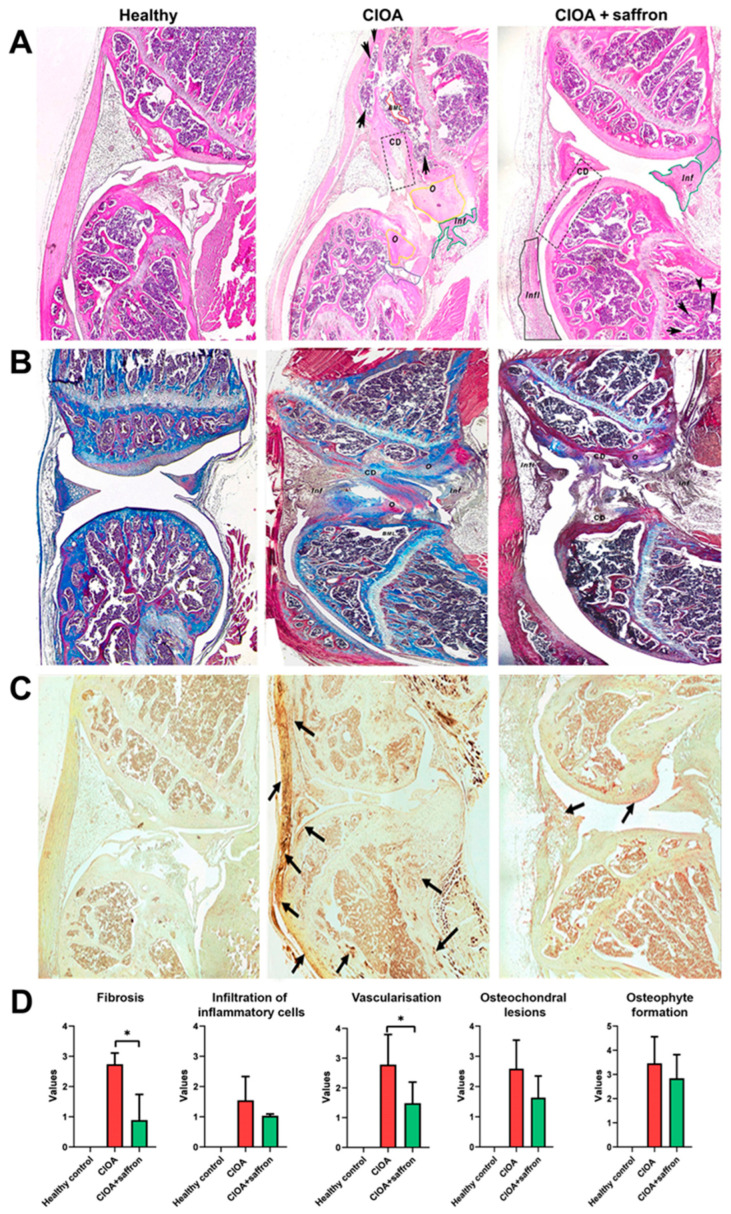
Histological analysis of the knee joint in groups of experimental animals with CIOA after 30 days of treatment with saffron extract (50 mg/kg). (**A**). Representative histological sections stained with Hematoxylin-Eosin; key pathological changes were observed and marked—osteophyte formation (O), cartilage destruction (CD), fibrous tissue deposition (Infl), cellular infiltration (Inf), partial bone marrow loss (BML) and adipocyte changes (arrows). (**B**) Representative histological sections stained with Trichrome Masson staining; the main pathological changes were observed and marked—osteophyte formation (O), cartilage destruction (CD), partial absence of bone marrow material (BML), fibrous tissue deposition (Infl), cellular infiltration (Inf). (**C**) Representative histological sections stained with Von Kossa staining; key pathological changes were observed and marked—subchondral bone erosion with osteoclast infiltration and bone destruction (black arrows). (**D**) Mean histopathological scores for fibrosis, synovium infiltration, vascularization, osteochondral lesions, and osteophyte formation. Data was quantified in a blinded manner. Results are representative of 5 animals analyzed. 5× original magnification. * *p* < 0.05.

Joints from healthy, untreated control animals showed normal synovial tissue (Sy), no detectable deformities in the femur, and no abnormalities in bone marrow structure (Figure 8A, left). In contrast, the CIOA animal group displayed significant pathological changes, including marked synovial inflammation, characterized by partial thickening of the synovial membrane with approximately seven layers of synoviocytes (Figure 8A, middle, Figure 8D). Prominent osteophyte formation was observed, with a large osteophyte at the edge of the femur and another between the tibia and femur in the area of the meniscus (O). Severe cartilage degradation (CD) was prominent, along with deformation of the tibial architecture, affecting both cartilage and subchondral bone. Marked growth plate (GP) hypertrophy and structural disorganization were also observed, along with subchondral bone marrow changes, including partial bone marrow loss (BML) and adipocyte transformation (arrows). In a group of CIOA mice treated with saffron, the joints showed mild synovial inflammation with deposition of fibrous tissue near the femoral cartilage (Infl) and localized infiltration of inflammatory cells at the distal end of the tibia (Inf) (Figure 8A, right, Figure 8D). Adipocyte changes were also present in the bone marrow (arrows). Although cartilage damage was observed in the tibia (CD), the severity was significantly reduced compared to the untreated CIOA group, suggesting a protective effect of saffron treatment.

Masson’s trichrome staining was used to assess structural integrity, fibrosis, and remodeling of the extracellular matrix (ECM) in articular cartilage and surrounding joint tissue in healthy and CIOA mice, with or without saffron extract treatment. The articular cartilage of the Healthy group appeared smooth and well-structured, with uniform collagen staining (blue) and minimal ECM disruption (Figure 8B, left). The subchondral bone and synovial tissue remained intact with no signs of fibrosis or inflammatory infiltration.

Significant disorganization of cartilage structure, erosion of the articular surface, and disruption of the tidemark were found in the CIOA group (Figure 8B, middle). The presence of marked collagen deposition (intense blue staining) in periarticular areas suggested fibrosis, while inflammatory cell infiltration was visible in the synovial membrane and joint capsule. Cartilage degradation (CD), fibrosis (Fib) and osteophyte formation (O) were also observed. In comparison, partial restoration of cartilage architecture and reduction in fibrous areas were found in saffron-treated CIOA mice compared to the untreated CIOA group (Figure 8B, right, Figure 8D). Collagen deposition was less pronounced, inflammatory infiltration (Inf) appears reduced, and although some degenerative changes persisted, overall histological damage was attenuated.

Von Kossa staining was performed for histological assessment of calcification. Intensified silver staining showed increased calcification, subchondral bone erosion, and osteoclast infiltration (black arrows) in the CIOA group (Figure 8C, middle). These findings defined severe bone destruction and mineral deposition, as well as bone remodeling dysfunction associated with CIOA. In contrast, animals from the saffron-treated CIOA group showed a significant reduction in calcification and attenuated bone damage, suggesting a protective effect of the extract in CIOA bone pathology (Figure 8C, right). As expected, no signs of pathological calcification or bone damage were observed in the Healthy group (Figure 8C, left).

### 2.7. Saffron Extract Enhances Nrf2 Expression in Synoviocytes Derived from Animals with CIOA

**Figure 9:**
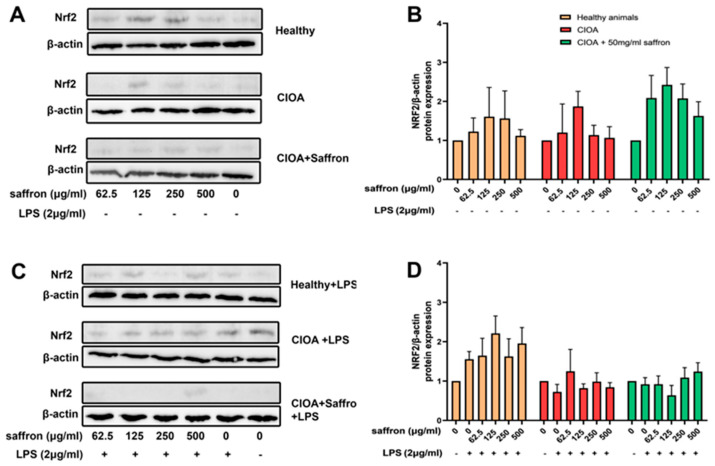
Effect of ex vivo treatment with saffron extract in the presence or absence of LPS (2 μg/mL) on intracellular Nrf2 expression in synoviocytes isolated from healthy mice and animals with CIOA. Representative images of Western blots depicting Nrf2 levels in synoviocytes (**A**,**C**) and protein/β-actin ratios determined by densitometry analysis of Western blots (**B**,**D**). Results are representative of 5 animals analyzed.

To investigate the therapeutic activity of saffron extract through Nrf2-mediated protective effects, we analyzed the protein expression of Nrf2 in synoviocytes from animals with CIOA treated or not with saffron extract. In vitro incubation of synoviocytes from healthy animals with increasing concentrations of saffron extract resulted in an increased Nrf2 expression at 125 µg/mL and 250 µg/mL of the studied extract (Figure 9A,B). Similarly, synoviocytes isolated from the CIOA + saffron group and treated in vitro with saffron extract showed more visible, dose-dependent upregulation of Nrf2 expression compared to cells from other experimental groups. In contrast, synoviocytes from control CIOA mice subjected to saffron extract in vitro showed lower Nrf2 expression compared to those from the CIOA + saffron group at all concentrations, except at 125 µg/mL.

LPS is a stressor that induces inflammatory responses, which often causes a secondary adaptive upregulation of transcription factors such as Nrf2. We used LPS as a control to demonstrate the activation of the Nrf2 pathway and to investigate the protective effects of saffron extract. In vitro co-stimulation with LPS did not increase Nrf2 expression overall in the two CIOA groups of mice compared to the healthy control group, where a moderate increase was recorded (Figure 9C,D). The results from both CIOA groups, co-stimulated in vitro with saffron extract and LPS, showed no effects on Nrf2 protein expression compared to the Healthy group.

## 3. Discussion

Osteoarthritis affects millions of people worldwide, causing pain, impaired mobility, and suffering for patients. This degenerative disorder involves articular cartilage, synovium, and subchondral bone. Pathological changes in affected joints during the progression of OA are associated with the formation of chondro/osteophytes, the development of synovitis, sclerosis of the subchondral bone, and loss of articular cartilage [50].

Natural extracts have been studied for years and have shown antimicrobial, antitumor, and pharmaceutical properties, mainly due to the large number of different bioactive compounds. *Crocus sativus* L. is a well-known plant, a perennial, stemless herb belonging to the family of Iridaceae. The distribution of crocus is wide, but the specific consistency of the active substances varies in the wild depending on the soil, climate, etc. Saffron was originally grown in Iran and cultivated in other countries such as France, Greece, Italy, Spain, India, China, Turkey, Lebanon, and Azerbaijan [51]. The phytochemical profile of saffron is well characterized. It contains 63% sugars, 12% proteins and amino acids, lipids, minerals, fibers, and vitamins. The bioactive compounds responsible for saffron’s main activities are flavonoids, carotenoids (crocin and crocetin), and terpenes (safranal). All of these compounds contribute to the antioxidant and anti-inflammatory effects of saffron. Crocin, a water-soluble carotenoid, has been extensively studied, exhibiting strong antioxidant effects by scavenging reactive oxygen species and enhancing the cellular antioxidant defense systems. Its potential has been shown in various mouse and rat models of neuroinflammation, arthritis, and asthma. Crocetin, a derivative of crocin, is known to modulate inflammatory signaling pathways, including inhibition of TNF-kB activation and production of downstream proinflammatory cytokines, such as TNF-α and IL-1β. Safranal, a major terpenoid compound, has been shown to have both anti-inflammatory and antioxidant properties, including regulation of apoptosis and mitochondrial function. These effects have been shown in neuronal studies, type -2 diabetes and diabetic nephropathy, as well as in neurodegenerative diseases [52].

Animal models used in OA research should display all important pathological features of the disease overlapping with human OA. Among them, collagenase-induced experimental OA generated in mice by intra-articular injection of collagenase showed osteophyte formation, cartilage erosions, osteoarthritic lesions, fibrosis, and activation of synovial macrophages. This experimental model provides observation of three phases of the disease—acute, active, and chronic phases of joint degeneration, which allows observation of induced inflammation and the release of pro-inflammatory factors into the synovial fluid [49].

We developed a collagenase-induced model of osteoarthritis (CIOA) in mice and characterized the immune factors responsible for inflammation and disease pathology. Immune cell phenotyping, as well as histological and synovial changes during CIOA progression, were analyzed by FACS and differentiation staining [49].

The specifics of natural extracts are related to the possibility of prolonged intake and toxic effects, based on well-known components and quantities. In our previous study, we investigated the effects of oral supplementation of saffron extract in mice with CIOA and the progression of the disease depending on the saffron concentration [53]. We found that the effectiveness of saffron therapy was dose-dependent, with the best results obtained at a concentration of 50 mg/kg. For this reason, we investigated more aspects of the effects of saffron extract on CIOA pathology and joint and muscle condition.

In this study, we used 50 mg/kg saffron extract for in vivo therapy of healthy mice and animals with CIOA, followed by a series of ex vivo experiments with isolated bone marrow and synovium cells and histological analyses of joints. The beneficial effect of the therapy on the level of inflammation and improved joint histology raises the logical questions about the potential mechanism for disease suppression. FACS analyses of some immune cell populations showed significant differences between untreated mice with CIOA and the group of sick animals to which saffron extract was administered in vivo. Some of the immune cells are directly involved in the pathological process of arthritis development, while other types of cells are involved indirectly through supportive functions. The bone marrow serves as a pool of reserve myeloid and lymphocyte cells, ensuring a balance between the different depots of immune cells. CIOA progression changed the ratio of different cell subpopulations within the two main cell types. Even if saffron extract treatment slightly altered the cell ratio of treated mice compared to healthy controls, the most notable increase after saffron therapy compared to the CIOA animal group was observed in patrolling monocytes (CD11b^+^ F4/80^−^Ly6C^low^) after 14 days of therapy. The same increase in these cells was found in the synovium also after 14 days of therapy, but without enhancement of M1 polarization at both sites. It is in contrast with the findings that Ly6C^−^ monocytes infiltrate the joint during the effector phase of arthritis and differentiate into pro-inflammatory M1 macrophages, thereby promoting joint pathology [20,54]. Interestingly, in line with this, another Ly6C^low^ cell population (CD11c^+^ F4/80^hi^) was significantly lower in the bone marrow after saffron therapy but dramatically increased in the synovium during the effector phase of CIOA.

Non-tissue resident CD11b^+^F4/80^−^Ly6C^+^ macrophages have the potential to regulate inflammation by differentiating into pro-inflammatory M1 or anti-inflammatory M2 phenotypes after homing to tissues [20]. No changes were found in the bone marrow after therapy between the groups, but a significant decrease and increase were measured in the synovium on day 7 and day 14, respectively. This dynamic may explain the positive effect of the therapy on the resolution of inflammation. Even if CD11b^+^F4/80^−^Ly6C^+^ macrophages are responsible for M1/M2 differentiation, no significant changes for M1 or M2 were found in either the bone marrow or the synovium, resulting from the synovial changes in CD11b^+^F4/80^−^Ly6C^+^ macrophages.

In addition, a significant decrease in the percentage of macrophage population (F4/80) was found in the joints of the saffron-treated group compared to untreated CIOA controls, which is an additional factor in the resolution of inflammation. No significant changes among the bone marrow cell population were found between any groups in the chronic phase of CIOA (day 30). More dynamics are observed in the chronic phase of CIOA for synovial cells, which relates to the importance of the therapeutic window and the timing of each therapy.

Neutrophils also participate in osteoarthritis pathophysiology, playing a complex role. Neutrophils are the first cells recruited into an inflamed joint and promote inflammation and joint degradation by producing pro-inflammatory cytokines and chemokines, reactive oxygen species (ROS), matrix-degrading enzymes, and neutrophil extracellular traps (NETs). The same cells are involved in joint repair by regulating the immune response and releasing anti-inflammatory factors [55]. Phenotyping of synovial cells of animals treated with saffron extract showed a significant reduction in the neutrophil population (CD11b^+^ Ly6G^+^) throughout the treatment period, while no such reduction was found in the bone marrow.

Lymphocytes play an important role in the progression of OA, and both T- and B-lymphocytes are involved in immune-induced inflammation and cartilage destruction within the synovial fluid of osteoarthritis joints. CD4^+^ T-lymphocytes produce IL-17, which can activate T-lymphocytes, mainly in the early stage of inflammation. IL-17 increases chondrocyte activity and induces cartilage degradation, promoting inflammation. Activated T cells produce IL-6, which has a dual effect, accelerating or suppressing the severity of osteoarthritis depending on several factors. Furthermore, IL-1β, TNF-α, and IL-6 contribute to the destruction of articular cartilage through several mechanisms [55].

Osteoblast maturation and activation are regulated by various immune cells, networked through complex mechanisms and involved mediators. CD4^+^ T lymphocytes play a major role and could promote the differentiation of bone marrow mesenchymal stem cells (MSC) through the secretion of IFN-γ, while osteoblast differentiation could be suppressed by TGF-β produced by the same cells. Furthermore, IL-17 may contribute to the induction of osteoblast differentiation from MSCs in bone. Regulatory T (Treg) cells can also stimulate MSC differentiation into osteoblasts through IL-17F, while B cells are enriched in the synovial microenvironment and can stimulate osteoclast formation through TNF-α-promoted RANKL secretion [56,57].

We did not observe significant changes among the populations of CD4^+^ and CD8^+^ T cells, as well as B cells, in the bone marrow between the experimental groups. Conversely, an increase in CD4^+^ and a decrease in CD8^+^ T cells were found in the synovium of mice with CIOA after saffron therapy. In line with the above discussion, these changes are likely related to the joint protection induced by saffron extract, but further detailed analyses of cell subpopulations may reveal the potential mechanism.

NK cells play a key role in the development and progression of OA, maintaining inflammation by interacting with chondrocytes, synoviocytes, osteoclasts, and other immune cells, determining the severity of the disease [58]. Here, even if the total populations of CD335+ NK cells were not altered in either the bone marrow or the synovium in both groups of mice with CIOA, a significant increase in mature CD27^−^CD11b^+^ NK cells was found after saffron therapy compared to control CIOA animals. This NK subset shows increased potential to promote osteoclast formation and reduced cytotoxicity and plays an important role in the pathogenesis of OA [59].

Combined analysis of bone marrow and synovium compartments indicated that saffron modulates the systemic-local inflammatory axis in CIOA. It limits the generation of pro-inflammatory myeloid cells in BM, reduces their recruitment into the synovium, and promotes a shift toward anti-inflammatory macrophage polarization and adaptive immune regulation within the joint. The observed effects suggest a multi-level immunomodulatory mechanism of action of the saffron extract. In the context of the saffron’s main components and their activities, crocin and crocetin could contribute to the reduction in oxidative stress within the joint microenvironment, reduce inflammatory cell infiltration, and alter immune cell dynamics in the synovium, thereby limiting cartilage degradation and synovial inflammation. Cell apoptosis also plays an important role in cartilage degeneration as part of the pathology of osteoarthritis. Normal cartilage in healthy mice shows low levels of apoptosis to remove damaged cells, while in OA, increased apoptosis maintains ongoing inflammation and contributes to cartilage breakdown [60,61]. The small number of cells isolated from the synovium did not allow us to perform an apoptosis assay, but we did so for splenocytes isolated from all experimental animals on days 7, 14, and 30 after OA induction. Analyzing B-cell apoptosis, we found a significant increase in live cells and a significant decrease in early apoptotic cells in the saffron-treated mice compared to the CIOA control group on day 30. The results show that B cells are less sensitive to inflammatory apoptosis. This was confirmed by the presented flow data—CD19^+^ populations remained relatively constant across the different groups. Comparison of the same groups of animals and analyses of T-cell apoptosis showed an improvement in overall T-cell survival and a significant reduction in late and early apoptosis at all control points after saffron administration. The increased apoptosis observed in CD3^+^ T cells in the CIOA group is consistent with the pro-inflammatory immune profile identified by flow cytometry. Inflammatory myeloid expansion is a source of inflammatory mediators capable of inducing T-cell apoptosis. Saffron treatment significantly reduced T-cell death and restored cell viability through modulation of the inflammatory microenvironment. By examining cell proliferation assays of splenocytes from all experimental groups after ex vivo stimulation with saffron, we found a dose-dependent increase in the proliferation index in both groups of CIOA mice relative to controls without ex vivo stimulation. Comparing the groups to each other, a significant suppression was found after ex vivo incubation with 1 mg/mL saffron extract in in vivo saffron-treated mice toward untreated animals with CIOA. Compared with the apoptosis data, these results indicate that saffron extract exerts cytoprotective rather than proliferative effects. Even if the spleen cannot accurately reflect the processes in the joints during the progression of OA, the influence of saffron extract therapy affects all reservoirs of immune cells, providing conditions for the resolution of inflammation, as well as attracting various cell types into the joint cavity.

Dysregulated function of both osteoclasts (bone-resorbing cells) and osteoblasts (bone-forming cells) in OA contributes to the disease’s progression, leading to abnormal bone remodeling and bone loss in the subchondral bone, caused by increased osteoclast activity and an imbalance in bone remodeling due to reduced or altered osteoblast function [62]. In our experiments, we found an in vivo and ex vivo effect of saffron extract therapy to limit pathological joint remodeling, due to suppression of bone-resorbing cell differentiation, with relevance only for in vivo therapy. As expected, osteoblast differentiation was suppressed in both groups of animals with CIOA, but ex vivo stimulation with saffron extract did not enhance the proliferation of bone-forming cells in mice treated with the extract in vivo. These findings are consistent with flow cytometry data showing reduced frequencies of Ly6C^+^ monocytes and inflammatory myeloid cells, which serve as precursors of osteoclasts. This framework was formed based on the stimulation of bone marrow cells, while effective protection can be observed in joint analyses. Indeed, when histopathological analyses were performed, the positive results of the therapy were observed, as the bone marrow destruction observed in animals with CIOA and treated with saffron extract was completely suppressed. The mild cartilage degeneration and lower levels of fibrosis in the CIOA + saffron group are likely due to inhibition of the invasion of inflammatory cells into the synovium and the compensatory mechanism to limit damage there, suggesting a protective effect of the therapy.

Nrf2, a transcription factor regulating antioxidant defense, is a key player in maintaining cellular homeostasis by regulating the transcription of intracellular gene factors associated with defense. Its protective effects have been recently demonstrated in a number of experimental models of chronic diseases such as diabetes, heart disease, and neurodegenerative diseases. Nrf2 plays a protective role in synoviocytes by reducing inflammation and oxidative stress, which are key features of OA. Notably, Nrf2 activation can suppress the production of pro-inflammatory cytokines and enhance the expression of antioxidant enzymes [63,64].

We investigated how saffron extract treatment can initiate signal transduction through Nrf2. In our hands, synoviocytes isolated from in vivo saffron extract-treated mice with induced CIOA showed dose-dependent elevated levels of Nrf2 expression after ex vivo stimulation with saffron extract. The density of immunoblot bands from this therapeutic group was much higher compared to untreated in vivo CIOA animals or control healthy animals. It appears that in vivo treatment with saffron extract alters the behavior of synoviocytes towards a protective phenotype and improves resistance to oxidative stress.

In the presence of LPS as an additional stimulation, Nrf2 expression was suppressed in both groups of CIOA animals compared to healthy controls, and ex vivo incubation of cells from the CIOA group treated in vivo with saffron failed to increase Nrf2 levels up to the healthy controls. As expected, the inflammatory stress of OA combined with LPS challenge, which induces additional oxidative reactions in cells, appeared to suppress Nrf2 levels, and treatment with saffron extract failed to increase them.

Therapy of mice with induced CIOA with 50 mg/kg saffron extract had a favorable effect on the course of the disease. Analysis of ex vivo experiments showed reduced pathohistological changes in the animal’s joints, preserved articular cartilage, reconstructed cellular properties and phenotype compared to the control group of mice. All of these findings demonstrate consistent immunomodulatory effects in systemic and local compartments in an experimental mouse model, but they require further validation in human OA.

## 4. Materials and Methods

### 4.1. Mice

Male BALB/C mice obtained from The Jackson Laboratory (Bar Harbor, ME, USA 04609) were housed in our barrier-type animal facility under specific-pathogen-free (SPF) conditions. Groups of 10–12-week-old animals were used for our experiments. The mice were randomly distributed to the respective groups (five animals per cage), and the maintenance and the manipulations were approved by the Animal Care Commission at the Institute of Microbiology in accordance with the international regulations (EU Directive 2010/63/EU) and with the permission of the Bulgarian Food Safety Agency (BFSA)-permit No. 306 (valid until 14 July 2026).

### 4.2. Antibodies

Anti-mouse Fluorescein isothiocyanate (FITC)-conjugated CD4 (clone GK1.5), CD8 (clone 53–5.7), CD11c (clone N418) and CD335 (clone 29A1.4); eFlour450–conjugated CD3 (clone 145-2C11); Phycoerythrin (PE)–conjugated F4/80 (clone 145-BM8), CD69 (clone H1.2F3), CD19 (clone 6D5) and CD 107a (LAMP-1) (clone 1D4B); Alexa Flour 700–conjugated Ly6C (clone HK1.4); Allophycocyanin (APC)–conjugated CD4 (clone GK1.5), CD25 (clone PC61), CD206 (clone C068C2) and CD11b (clone M1/70); Pacific Blue conjugated Ly6G (clone 1A8) and CD80 (clone 16-10A1) (BioLegend, Amsterdam, The Netherlands) were used for Fluorescence-activated cell sorting (FACS) experiments. Relevant Isotype-matched control IgG antibodies (BioLegend, Amsterdam, The Netherlands) were used for compensation and primary antibody staining validation. Pacific blue-conjugated anti-mouse CD19 antibody (clone 6D5, BioLegend, Amsterdam, The Netherlands) and Pe Cy7-conjugated anti-mouse CD3 antibody (clone 145-2C11, BioLegend, Amsterdam, The Netherlands) were used for apoptosis assay.

### 4.3. Induction of Osteoarthritis

The mouse model of collagenase-induced arthritis (CIOA) was developed as previously described with slight modifications [49,50]. Briefly, animals were anesthetized, and an intra-articular (i.a.) injection of 10 µL/knee Collagenase type IA (2 mg/mL, from Clostridium histolyticum, Sigma-Aldrich, Darmstadt, Germany, #C9891) was performed in both knees.

### 4.4. Crocus Sativus Extract Preparation

Saffron extract was prepared as described before [53]. The alcoholic extract of saffron used in the current study was kindly provided by Prof. Ulduz Hashimova, Garayev Institute of Physiology of Azerbaijan National Academy of Sciences, Azerbaijan. The preparation of the extract was described previously [53]. Briefly, the tested extract was obtained by evaporation of a 75% ethanolic solution containing 5g of dried saffron stigmas, followed by vacuum concentration. A portion of the lyophilized extract was used for NMR and HPLC analyses.

For metabolite profiling of the tested component, 1H NMR and 2D NMR spectroscopy (J-resolved, HSQC and COSY) were performed using an AVII+ 600 spectrometer (Bruker, Karlsruhe, Germany). After automatic conversion of the spectra into ASCII files using AMIX software (version 3.7, Bruker), phase- and baseline-correction was performed using MestReNova software (version 12.0.0, Mestrelab Research, Santiago de Compostela, Spain). The signals were normalized according to the peak of TSPA-d4 and scaled to 1.0 [53].

The analysis of the major secondary metabolites was carried out by HPLC UV-VIS using a Waters HPLC system. Quantification of picrocrocin, crocin 1, crocin 2, and safranal was performed according to [53]. The extract and the reference standards were analyzed using gradient elution systems with aqueous formic acid/methanol or water/acetonitrile as mobile phases. Detection wavelengths were set at 440 nm for crocin 1 and crocin 2, 250 nm for picrocrocin, and 303 nm for safranal. The validation included assessment of linearity, limit of detection (LOD), and limit of quantification (LOQ) using external calibration curves.

### 4.5. Treatment Schedule

The day after osteoarthritis induction, the animals were randomized into three groups. A group of animals injected with collagenase was treated daily per os with 50 mg/kg saffron extract (CIOA + saffron). Two control groups, one with animals injected with collagenase (CIOA group) and another with healthy Balb/c mice, received daily per os 300 µL PBS (healthy control).

### 4.6. Isolation of Splenocytes

The experimental animals were sacrificed, and the spleens were taken 30 days after the induction of arthritis. Single cell suspensions were prepared by mechanical grinding, and splenocytes were passed through cell strainers (BD Biosciences, Erembodegem, Belgium). Cell suspensions were centrifuged at 1300 rpm for 10 min at 4 °C, and erythrocytes were lysed with red blood cell (RBC) lysis buffer (150 mM NH_4_Cl, 10 mM KHCO_3_, 0.1 mM Na_2_EDTA, pH 7.2) for 5 min at RT. After washing with PBS, the supernatants were discarded, and the cell pellets were homogenized in RPMI (Roswell Park Memorial Institute) 1640 medium (GE Healthcare, Hatfield, UK) and counted for further analyses.

### 4.7. Isolation of Bone Marrow Cells

Bone marrow cells were isolated from a mouse femur. The femur was separated from the hip joint under sterile conditions, and after removal of soft tissues, the bones were placed in a culture dish with sterile PBS. Both ends of the bone were cut off, and a 23-gauge needle and syringe filled with RPMI were used to flush the bone marrow onto a 70 μm cell strainer (BD Biosciences) placed in a sterile cell culture dish. The bone marrow was smashed through the cell strainer with a plunger and washed with RPMI. Cell suspensions were centrifuged at 1300 rpm for 10 min at 4 °C, supernatants were discarded, and cell pellets were homogenized in 1 mL of RBC lysis buffer. After 1–2 min of incubation at room temperature, the cells were washed, counted with a hemocytometer, and used for flow cytometry analysis or for osteoclast and osteoblast differentiation.

### 4.8. Preparation of Synovial Cells

Synovium was isolated as described by Armaka et al. [65]. Briefly, after removal of soft tissues, the hind legs were dissociated, and the intra-articular synovium was collected by dissecting the knee joint cavity. Isolated tissues were incubated in filtered (0.22 μm) freshly prepared collagenase II solution (1 mg/mL) in Dulbecco’s Modified Eagle Medium (DMEM) supplemented with 10% Fetal Calf Serum (FCS), 1% L-glutamine and antibiotics. The hind legs were incubated with collagenase solution at 37 °C for 1 h with shaking. The bones were then vortexed vigorously to release the cells. The supernatants were transferred to new tubes and fresh DMEM was added to the digested joints and vortexed again. The collected supernatants were centrifuged at 1100 rpm for 10 min at room temperature. Cell pellets were homogenized in fresh DMEM and passed through a 70 μm cell strainer. The resulting synovial cells were counted and used for flow cytometry analysis.

### 4.9. Flow Cytometry Analysis

The cells isolated from BM and synovium (2 × 10^6^ cells/mL) were washed with PBS (containing 2.5% FCS and 0.05% sodium azide) and incubated with matching anti-mouse antibodies. Several mixtures of anti-mouse antibodies were used to gate the following cell types: Myeloid lineage: CD11b-APC for myeloid cells; Ly6G-Pacific Blue for Neutrophils; Ly6C-AlexaFlour 700, and F4/80-PE for Inflammatory monocytes, Patrolling monocytes and Macrophages; F4/80-PE, CD80- Pacific Blue, and CD206-APC for M1/M2 macrophages; Lymphocyte lineage: CD19-Pacific Blue for B-lymphocytes; CD3-PeCy7, CD4-APC, and CD8-FITC for T-lymphocytes; CD3-Pacific Blue, CD335-FITC, CD11b-APC, and CD27-PE for NK cells. Each incubation step was performed for 20 min on ice. Thirty thousand cells were analyzed from each sample with a BD LSR II flow cytometer using the FlowJo (v.10) software (BD Biosciences, San Jose, CA).

### 4.10. MTT Assay

Spleen cells isolated 30 days after the induction of arthritis (2 × 10^6^ cells/mL) from all experimental groups were incubated with different concentrations of *Crocus sativus* extract for 48 and 72 h at 37 °C, 5% CO_2_. Control cells were stimulated with lipopolysaccharide (LPS, 10 µg/mL; Sigma-Aldrich). Four hours before the end of the incubation time, 5 mg/mL 3-(4,5-dimethylthiazol-2-yl)-2,5-diphenyltetrazolium bromide (MTT) was added to each well. Dimethyl sulfoxide (DMSO) was added to dissolve the formazan crystals, and the absorbance of the colored solution was measured at 590 nm.

### 4.11. Apoptosis

To analyze ex vivo the effect of saffron extract treatment on apoptosis levels at day 7, day 14, and day 30 after osteoarthritis induction, splenocytes (2 × 10^5^ cells/sample) from all experimental groups were stained with Pacific blue-conjugated anti-mouse CD19 and PeCy7-conjugated anti-mouse CD3 antibodies. Cell apoptosis was assessed with the Annexin V-FITC Apoptosis Detection Kit (BMS500FI-300, eBioscience, San Diego, CA, USA) according to the manufacturer’s instructions. Thirty thousand cells were analyzed from each sample with a BD LSR II flow cytometer using Diva 6.1.1 software (BD Biosciences, San Jose, CA, USA).

### 4.12. Osteoclasts Differentiation

Isolated bone marrow cells (2 × 10^6^ cells/mL) were counted and cultivated in 24- or 48-well cell culture plates. Different concentrations (0.25 mg/mL, 0.5 mg/mL, 1 mg/mL, 1.5 mg/mL, 2 mg/mL, 5 mg/mL) of the studied extracts were added to the respective wells. Some of the cells were incubated with an additional stimulus, 1,25-dihydroxyvitamin D3 (1,25(OH)2D3), which induced RANKL expression and differentiation of the isolates into osteoclasts [66]. Cells were cultured for 7 days in RPMI medium containing 10% FCS, 4 mM L-glutamine, 50 mM 2-mercaptoethanol and antibiotics. The medium and stimuli were changed every three days, and after the seventh day, the cells were stained with crystal violet.

### 4.13. Osteoblasts Differentiation

Bone marrow cells (2 × 10^6^ cells/mL) were cultivated in 24- or 48-well plates for 14 days in MEM culture medium (Minimum Essential Medium; Gibco, Gaithersburg, MD, USA), supplemented with 10% FCS, 4 Mm L-glutamine, 50 Mm 2-mercaptoethanyl and antibiotics in the presence of increasing concentrations of saffron extract (0.25 mg/mL, 0.5 mg/mL, 1 mg/mL, 1.5 mg/mL, 2 mg/mL, 5 mg/mL). Some of the cells were stimulated with vitamin C and β-Glycerophosphate disodium salt hydrate (Sigma-Aldrich; #G9422) for osteoblast differentiation [67]. The medium and the stimuli were changed every three days with new ones. At the end of the culture period, the cells were stained using the Von Kossa method.

### 4.14. Histological Analysis

At least 5 animals per group were processed for histological analysis. The knee joints were separated, fixed in 10% phosphate-buffered formalin (pH 7.4) for 5 days, and subsequently decalcified in 20% EDTA for 10 days. After automated tissue processing (TP1020 from Leica Biosystems, Germany), bones were embedded in paraffin (Paraplast Plus^®^, Sigma-Aldrich), then cut into sections (5–7 mm) and stained with hematoxylin and eosin (H&E) for general morphology observation. Specific staining with Masson’s trichrome was performed to confirm pathological changes in the knee. The OARSI Osteoarthritis Cartilage Histopathology Assessment System (scores between 0 and 4) was used to score the degree of histopathology alterations and to evaluate the effects of saffron extract [49,68]. Histological evaluation was performed in a blinded manner by an independent observer who was unaware of the experimental groups.

### 4.15. Western Blotting

Primary mouse synovial cultures isolated from all experimental groups, as described above, were used to assess nuclear factor erythroid 2-related factor 2 (NRF2) protein expression by Western blotting. Synoviocytes obtained between passages 3 and 5 were cultured in a 6-well plate (1 × 10^6^ cells/mL) with different concentrations of the saffron extract (62.5 µg/mL; 125 µg/mL; 250 µg/mL; 500 µg/mL) for 24 h at 37 °C/5% CO_2_. Half of the samples were stimulated with 2 μg/mL LPS for an additional 24 h. Nuclear and cytoplasmic protein lysates were then prepared using RIPA buffer (10 mM Tris base pH 7.4, 50 mM NaCl, 1% Triton X- 100) supplemented with protease and phosphatase inhibitor cocktails (1 mM sodium orthovanadate; 50 mM NaF; 25 mM sodium pyrophosphate; 5 mM EDTA; Protease inhibitor cocktail 100x).

Bradford assay (Sigma-Aldrich, #B6916) was used to measure the protein levels, and equal amounts of protein were loaded onto 10% Sodium dodecyl sulfate (SDS)- Polyacrylamide gel electrophoresis (PAGE) and transferred to polyvinylidene difluoride (PVDF) membrane (Immobilon^®^-P membrane, Merck, Darmstadt, Germany). The membrane was blocked with 5% skim milk in Tween-Tris-buffered saline (TBS) for 1 h at RT. Next, the membrane was blotted overnight at 4 °C with anti-Nuclear erythroid-2-related factor 2 (Nrf2) (1:1000, clone D1Z9C; #12721, Cell Signaling, Danvers, MA, USA), and anti- β-actin (1:1000, clone 13E5; #4970, Cell Signaling) rabbit antibodies. After washing with Tween-TBS, the membrane was incubated with secondary HRP-conjugated anti-rabbit antibody (1:1000, #7074, Cell Signaling) for 1 h at RT. Samples were detected with enhanced chemiluminescence (ECL) (Pierce™ ECL Western blotting Substrate; #32106, Thermo Fisher Scientific, Waltham, MA, USA), and evaluated by Image system (Bio-Rad ChemiDoc, CA, USA). The developed blots were analyzed by densitometry using ImageJ Software (version 1.54g).

### 4.16. Statistical Analysis

Data are presented as mean ± SD and compared by two-way ANOVA with Tukey’s post hoc test. In all experiments, a *p*-value < 0.05 was considered significant. All statistical analyses were done with GraphPad Prism version 9 (GraphPad Prism software Inc., San Diego, CA, USA).

## Data Availability

The original contributions presented in this study are included in the article. Further inquiries can be directed to the corresponding author.

## Abbreviations

The following abbreviations are used in this manuscript:

OA	Osteoarthritis
NK	natural killer cells
DCs	dendritic cells
NSAIDs	nonsteroidal anti-inflammatory drugs
SNRIs	serotonin-norepinephrine reuptake inhibitors
SPF	specific-pathogen-free conditions
FITC	Fluorescein isothiocyanate
APC	Allophycocyanin
FACS	Fluorescence-activated cell sorting
CIA	collagenase-induced arthritis
i.a.	intra-articular
RBC	red blood cell
RPMI	Roswell Park Memorial Institute
DMEM	Dulbecco’s Modified Eagle Medium
FCS	Fetal Calf Serum
LPS	lipopolysaccharide
MTT	3-(4,5-dimethylthiazol-2-yl)-2,5-diphenyltetrazolium bromide
DMSO	Dimethyl sulfoxide
NRF2	nuclear factor erythroid 2-related factor 2
PVDF	polyvinylidene difluoride
